# Waning in influenza vaccine effectiveness against influenza A(H1N1)pdm09-associated hospitalization in children in 2012/2013

**DOI:** 10.1017/S0950268825100770

**Published:** 2025-11-24

**Authors:** Hui Ying Chua, Tim K. Tsang, So-Lun Lee, Eunice L.Y. Chan, Mike Y.W. Kwan, Joshua S.C. Wong, Malik Peiris, Sheena G. Sullivan, Benjamin J. Cowling

**Affiliations:** 1 WHO Collaborating Centre for Infectious Disease Epidemiology and Control, School of Public Health, Li Ka Shing Faculty of Medicine, The University of Hong Kong, Pokfulam, Hong Kong Special Administrative Region, China; 2 Laboratory of Data Discovery for Health Limited, Hong Kong Science and Technology Park, New Territories, Hong Kong Special Administrative Region, China; 3 Department of Paediatrics and Adolescent Medicine, Queen Mary Hospital and Li Ka Shing Faculty of Medicine, The University of Hong Kong, Hong Kong Special Administrative Region, China; 4 Department of Paediatrics and Adolescent Medicine, Princess Margaret Hospital, Hong Kong Special Administrative Region, Chin; 5 School of Clinical Sciences, Monash University, Melbourne, Australia; 6 Department of Epidemiology, University of California, Los Angeles, USA

**Keywords:** vaccine effectiveness, influenza, depletion-of-susceptibles

## Abstract

Measuring waning in vaccine effectiveness (VE) is challenging due to potential depletion-of-susceptibles bias. Some SARS-CoV-2 studies excluded individuals with prior infection and adjusted for the probability of remaining uninfected. We applied this approach to assess waning influenza VE in Hong Kong during the 2012/2013 season. First, we estimated the infection risk for unvaccinated children using published serological and surveillance data. Next, we derived infection risk for vaccinated children, assuming VE against infection of 57%. Uncorrected VE from 14 to 270 days post-vaccination was estimated from hospitalized children. We calculated the rate of depletion of susceptibles given infection risk and VE corrected for depletion-of-susceptibles bias. Waning rates for uncorrected and bias-corrected VE were measured by comparing VE at day 270 versus day 14. Bias was assessed as the absolute difference between two waning rates in percentage points. Waning rate of uncorrected VE was overestimated by 5.9 percentage points or 1.3 percentage points when assessed up to day 120. Bias was substantial when assuming 80% unvaccinated, and all vaccinated children were initially uninfected, but minimal when these proportions were similar. The observed waning in 2012/2013 was unlikely due to depletion-of-susceptibles bias. Further studies across various conditions are needed to confirm our findings.

## Key results and their importance

- We quantified bias due to depletion of the susceptibles in influenza vaccine effectiveness over time by correcting for the probability of remaining uninfected given vaccination status.
- Depletion-of-susceptibles bias was likely to have minimal impact on our observed waning estimates.
- Waning VE remains a problem, and optimizing the timing of vaccination will allow maximal public health impact of influenza vaccination campaigns.

## Introduction

Influenza vaccination provides children with effective protection against influenza-associated hospitalization. However, the protection provided by vaccination gradually wanes over time, and annual revaccination is recommended. In addition, circulating influenza viruses evolve antigenically necessitating annual updates to the recommended vaccine composition. Our group [1] and others [2, 3] have attempted to estimate the rate at which protection wanes over time. However, these previous analyses often did not account for differential depletion of susceptible individuals through acquisition of immunity from natural infection [4]. When vaccination is effective, the unvaccinated are at higher risk of infection than the vaccinated. As the epidemic progresses, however, the unvaccinated group becomes depleted of susceptible individuals more quickly than the vaccinated group, resulting in an apparent decrease in VE at later stages of the epidemic, irrespective of any true waning of protection [5].

To address potential bias due to the differential depletion of susceptible individuals, Ray et al. proposed a cohort variant of the commonly used test-negative design (TND) by restricting the study to vaccinated individuals [4, 6]. A limitation of this approach is that it can only be used to estimate relative, not absolute, VE. Andrejko et al. [7] estimated the degree of waning VE for SARS-CoV-2 in California, excluding individuals with recent prior SARS-CoV-2 infection. Recognizing the limitations of this approach (e.g., not all prior infections are recognized), they additionally proposed a method incorporating information from a state-wide seroprevalence survey to adjust for the probability of remaining uninfected given vaccination status. Our current study aims to apply that model for influenza VE.

In Hong Kong, influenza vaccination is currently prioritized in high-risk groups, including elderly above 50 years of age, children between 6 months and 18 years of age, and individuals with chronic illnesses [8]. Previous work measuring influenza VE over time in children from a long-standing hospital-based TND in Hong Kong estimated that influenza VE wanes by 2 to 5 percentage points per month [1]. Here, we aim to investigate the extent to which this observed waning in influenza VE may be attributed to differential depletion-of-susceptibles bias using the approach proposed by Andrejko et al. [7]. As with SARS-CoV-2, many influenza virus infections are mild and often unidentified. We, therefore, incorporated serological data from an independent longitudinal study to adjust for the probability of remaining uninfected and re-estimated waning in influenza VE.

## Methods

We analysed data from two existing studies and local surveillance to estimate VE against hospitalization due to influenza A(H1N1)pdm09, corrected for depletion-of-susceptibles bias, during the 2012/2013 influenza season (2 September 2012 to 31 May 2013) when influenza A(H1N1)pdm09 predominated. We pooled data from 2012/2013 to 2016/2017 to improve VE estimation. Most circulating strains of influenza A(H1N1)pdm09 during this study period were antigenically similar to the vaccine component A/California/7/2009 (H1N1)-like virus, enabling us to infer VE for the 2012/2013 season using pooled data.

### TND data

We used data from an existing TND study of influenza VE in two Hong Kong public hospitals to estimate VE [1]. Children aged 6 months to 17 years admitted for acute respiratory illnesses (i.e., fever ( 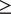 38 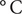 ) plus any respiratory symptom, such as cough, runny nose, or sore throat) were enrolled. Demographic and vaccination details were obtained by interviewing parents or legal guardians using standardized questionnaires. Vaccination status and date of vaccination were subsequently verified through electronic medical records, through vaccination cards, or by contacting private clinics where vaccines were administered. Children were considered vaccinated for the current season if they received an appropriate number of vaccine doses after 1 August each season and 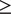 14 days before hospital admission. We excluded children with missing RT-PCR results, vaccination status, or time of vaccination and those who required two vaccine doses but received only one or were vaccinated within 14 days of admission. Time since vaccination for vaccinated children was calculated in days from the date of vaccination to hospital admission, while coded as 0 for unvaccinated children. Nasopharyngeal aspirates were collected and tested for influenza A and B using direct immunofluorescence assay and RT-PCR [9]. We defined cases as children testing positive for influenza A(H1N1)pdm09 and controls as children testing negative for all influenza types/subtypes. This VE study received ethical approval from the Institutional Review Board of the University of Hong Kong and the Hong Kong Hospital Authority for the West and East Clusters Research Ethics Committees.

### Serological data

We estimated the daily infection risk in unvaccinated children in the population during the study period using published serological data for the period between year 2009 to2013 [10, 11]. Six consecutive epidemics occurred during this period, with three predominated by influenza A(H1N1)pdm09 and three by influenza A(H3N2). The serological data came from a household study which commenced in 2009, recruiting households with 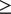 1 child aged 6 to17 years. One eligible child from each household was randomized to receive either influenza vaccination or saline placebo [12]. Serum samples were collected pre-vaccination and a month post-vaccination from August 2009 to February 2010. Households were followed from 2010 to 2013 during which receipt of annual influenza vaccination was observed, not randomized, and serum samples were collected twice annually during autumn (October to December) and spring (April to May) [11]. Serum samples were tested for anti-influenza antibodies using the haemagglutination inhibition (HAI) assay [13]. In this analysis, only HAI titres against A/California/7/2009-(H1N1) were considered.

Influenza infections were identified through active, fortnightly monitoring for acute upper respiratory tract infections (URTIs), defined as at least two symptoms: fever ( 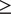 37.8 °C), chills, headache, sore throat, cough, presence of phlegm, coryza, or myalgia. Upon reporting a URTI, serum samples and combined nose and throat swabs were collected from all household members and tested by RT-PCR to identify influenza virus infections. This community-based cohort study received ethical approval from the Institutional Review Board of the University of Hong Kong.

### Influenza-like illness surveillance data

Influenza activity in the local community was monitored through a local sentinel surveillance network, with data obtained from the Centre for Health Protection website to inform the timing of infection for our infection risk estimation [14]. An incidence proxy was calculated by multiplying the outpatient influenza-like illness consultation rate among all consultations with the proportion of influenza A(H1N1)pdm09-positive specimens detected among all specimens tested for influenza viruses [15].

### Overview of statistical analysis

We gathered data from the TND studies, household serological studies, and surveillance network to estimate VE corrected for depletion-of-susceptibles bias using the equation [7]:
(1)

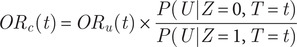

In this equation, 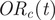 represents the odds ratio for bias-corrected VE at 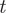 days post-vaccination, while 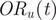 represents the odds ratio for uncorrected VE at 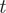 days post-vaccination. 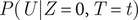 and 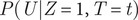 are the probabilities of remaining uninfected based on vaccination status, with 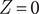 for unvaccinated individuals and 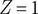 for individuals vaccinated 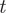 days prior.

The probability of remaining uninfected among vaccinated children is expressed as follows [7]:
(2)

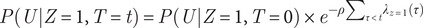

Here, 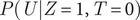 indicates the proportion of vaccinated children who remained uninfected during the current season at 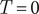 , that is, at the time of vaccination. The term 
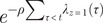
 represents the cumulative daily incidence risk of infection during days 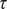 from 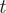 days since vaccination to testing. The parameter 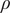 serves as a multiplier to correct for any underestimation of the cumulative daily incidence risk.

Similarly, for unvaccinated children, the probability of remaining uninfected is expressed as follows:
(3)

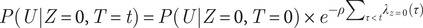

Here, 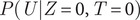 represents the proportion of unvaccinated children who remained uninfected during the current season at 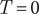 , when vaccination was offered (and would be accepted by the vaccinated children under comparison). The term 
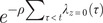
 represents the cumulative daily incidence risk of infection among those unvaccinated, the underestimation of which can also be corrected by 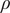 .

We first estimated the cumulative daily infection risk for unvaccinated and vaccinated children from 2 September 2012 to 31 May 2013, using the terms 
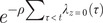
 and 
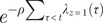
 . This estimation was based on serological and surveillance data, assuming children participated in the serological study were representative of children enrolled in the TND study. Next, we estimated 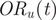 , the odds ratio for uncorrected VE against hospitalization due to influenza A(H1N1)pdm09, using TND data. Finally, we calculated the depletion of susceptibles given infection risk and estimated 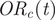 , reflecting VE corrected for depletion-of-susceptibles bias in a hypothetical cohort. The following subsections detail these steps.

### Estimation of infection risk in unvaccinated and vaccinated children

The depletion of susceptible children in the TND study was estimated from daily infection risks among unvaccinated and vaccinated children. We first estimated infection risk in unvaccinated children using a multilevel hierarchical Bayesian model developed by Tsang et al. [10, 16]. This model uses pre-epidemic, mid-epidemic, and post-epidemic HAI titre data from unvaccinated children and local surveillance data to reconstruct unobserved individual-level HAI titre trajectories for estimating infection risk.

The multilevel hierarchical Bayesian model incorporates a measurement model, HAI titre dynamics model, and infection model. The measurement model modelled underlying true HAI titre, which was unobserved, on a continuous scale. It assumes that where the true HAI titre falls within intervals between titre T and T + 1 dilution levels of the HAI assay, the observed titre would be measured as T in the absence of measurement error. The probability of measurement error was estimated as described by Cauchemez et al. [17]. The HAI titre dynamics model assumes infection boosts HAI titre within 14 days and HAI titre wanes thereafter. Both boosting and waning rates were modelled using Gamma ( 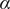 , 1) distributions where corresponding shape parameters 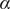 were estimated. The infection model estimates daily infection risk in children by multiplying the incidence proxy from surveillance data by a scaling factor 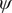 , and pre-epidemic titre was considered as a covariate associated with infection risk.

The unobserved HAI titre trajectory was sampled using a Bayesian data augmentation framework with a reversible-jump Markov Chain Monte Carlo (MCMC) algorithm to infer infection status, infection time, boosting, and waning parameters for each individual [10]. At each MCMC iteration, individual infection status was added or removed as informed by the dynamics of HAI titre and infection risk, and parameters were updated conditional on augmented infection status. We ran the algorithm on five independent chains of 4,000 iterations each, including a 2,000-iteration warm-up. The model, stratified by influenza A subtype, allowed us to obtain estimates for influenza A(H1N1)pdm09 in 2012/2013 without relying on the fourfold seroconversion criterion or PCR status to determine infection status, which could underestimate infections by 23% to 59% [10]. This approach effectively minimizes the risk of underestimating infection risks. See reference [10], for further information and validation of the modelling approach.

After estimating infection risk for unvaccinated children, we calculated infection risk for vaccinated children by multiplying the infection risk among unvaccinated children with the odds ratio (OR) for VE against A(H1N1)pdm09 infection among children, assumed to be 57% (OR = 0.43) based on estimates from a published TND study [18]. In sensitivity analyses, we examined our results assuming VE of 30% or 70% [18, 19].

### Estimation of uncorrected VE

We estimated VE from the odds ratio comparing the odds of vaccination among test-positive cases to test-negative controls. We referred to this as ‘uncorrected VE’ and denoted the corresponding odds ratio as: 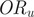 . Uncorrected VE was calculated as follows:



(4)

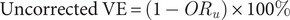

To estimate 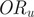 , we employed a conditional logistic regression model:
(5)

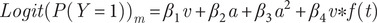

Here, 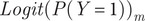 represents the log-odds of test-positive cases for the calendar month of admission 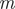 , where 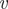 represents vaccination status, 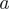 represents age, 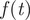 represents time since vaccination, and 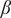 represents coefficients associated with each variable. The model was fitted without an intercept, matched by calendar month of admission, and adjusted for age and age-squared to address potential non-linear effects of age [20]. We included an interaction term between vaccination status and time since vaccination to model changes in vaccine protection [7].

The two main sources of confounding in VE studies are age and calendar time. We mitigated confounding from age by restricting the sample to children and further adjusting for age. Confounding due to time was addressed through matching. We compared models incorporating time since vaccination as linear, square root, logarithmic, and square functions using leave-one-out cross-validation to select the best-fitting model [21].

We implemented our model in a Bayesian framework using the R package ‘rstan’, running five independent chains of 4,000 iterations each, including a 2,000-iteration warmup. Based on model output, we predicted 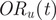 by varying 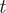 from 14 to 270 days post-vaccination and calculated uncorrected VE over this period 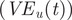 . We estimated the waning rate of uncorrected VE 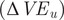 to quantify waning over 9 months post-vaccination, calculated as follows:
(6)

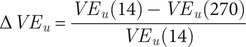

where 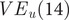 and 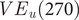 represent uncorrected VE at 14 and 270 days post-vaccination. In our main results, we also predicted 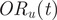 and 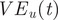 for 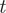 varying from 14 to 120 days post-vaccination and calculated 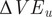 to quantify waning over 4 months post-vaccination.

### Estimation of bias-corrected VE

We estimated VE corrected for potential bias due to depletion of susceptibles using the method proposed by Andrejko et al. [7]:
(7)

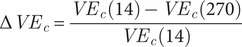

Here, 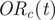 denotes the odds ratio corresponding to bias-corrected VE at 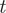 days post-vaccination, while 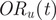 is the odds ratio from the conditional logistic regression model estimating uncorrected VE at 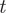 days post-vaccination. 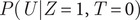 indicates the proportion of vaccinated children who remained uninfected during the current season at the time of vaccination 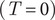 . The term 
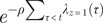
 represents the cumulative daily incidence risk of infection during days 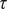 from 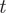 days since vaccination to testing among vaccinated children, and 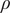 corrects for any underestimation of this risk.

Similarly, 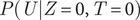 represents the proportion of unvaccinated children remaining uninfected for the current season at 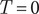 , when vaccination was offered (and would have been accepted by the vaccinated children in comparison). The term 
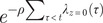
 represents the cumulative daily incidence risk of infection among those unvaccinated, the underestimation of which from the serological model can also be corrected by 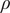 .

In primary analyses, we assumed that all children were uninfected for the current season at: 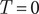 , that is, 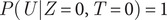 and 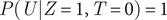 . While prior infection or vaccination may confer some level of protection against circulating viruses in the current season, we assumed equal chances of infection due to waning immunity. In sensitivity analyses, we set the proportion of unvaccinated children remaining uninfected at 0.8, while assuming all vaccinated children remained uninfected at 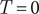 , and vice versa. We further varied these proportions between 0.50 and 1.0. In primary analyses, we set 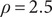 to address potential underestimation of infection risk. We also simulated scenarios with 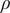 = 1.0 for accurate estimation and 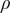 = 5 for more substantial underestimation of infection risk. Based on these assumptions, we calculated 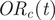 by varying 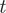 from 14 to 270 days post-vaccination and derived bias-corrected VE as follows:



(8)

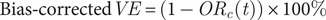

The waning rate for bias-corrected VE, 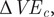 was calculated as follows:
(9)

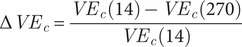

where 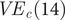 and 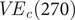 represent bias-corrected VE at 14 and 270 days post-vaccination. In our main results, we also estimated 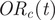 and 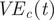 at 14 and 120 days post-vaccination, along with corresponding 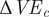 .

Depletion-of-susceptibles bias was assessed by comparing the waning rate of uncorrected VE 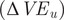 and waning rate of bias-corrected VE 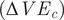 over 9 months post-vaccination. In our main results, we also evaluated potential bias in the waning rate of uncorrected VE over 4 months by comparing it to the waning rate of bias-corrected VE during the same period. An absolute difference of 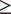 10 percentage points between the two waning rates 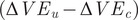 was considered significant. All analyses were conducted using R version 4.3.1 (R Foundation for Statistical Computing, Vienna, Austria).

## Results

**Figure 1.**
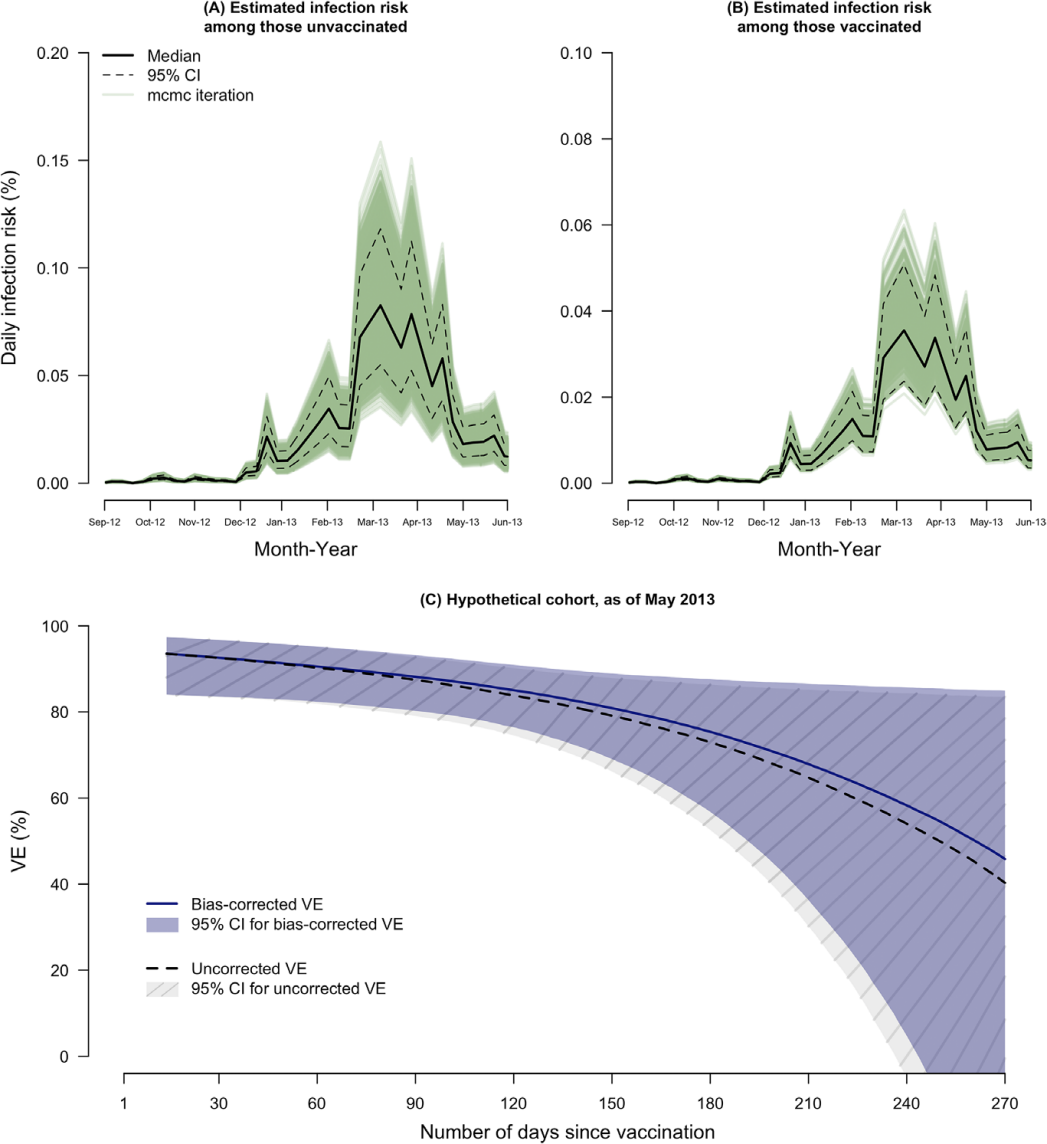
Estimated daily infection risk among (A) unvaccinated and (B) vaccinated children from September 2012 to the end of May 2013. (C) Uncorrected (dashed line) and bias-corrected (solid line) VE against hospitalization due to influenza A(H1N1)pdm09 by number of days since vaccination in a hypothetical cohort, as of May 2013. Note: grey area with patterns indicates 95% credible intervals (CI) of uncorrected VE and blue area indicates 95% CI of bias-corrected VE.

By fitting our multilevel hierarchical Bayesian model to serological data from 995 unvaccinated children recruited from 678 households, we estimated daily infection risk from 2 September 2012 to 31 May 2013. Influenza activity peaked in March 2013, with an estimated infection risk of 0.08% (95% confidence interval, CI: 0.06%, 0.12%) in unvaccinated children (Figure 1A). Assuming VE against influenza A(H1N1)pdm09 infection of 57%, we estimated daily infection risk in vaccinated children peaked at 0.04% (95% CI: 0.02%, 0.05%) (Figure 1B).

A total of 13,570 children (mean age = 3.6 years) enrolled in the TND study were included in VE estimation. The conditional logistic regression model incorporating time since vaccination as a linear function outperformed models with square root, square, or logarithmic functions (data not shown). Uncorrected VE against hospitalization due to influenza A(H1N1)pdm09 waned from 93.5% (95% CI: 84.0, 97.3) at 14 days to 40.3% (95% CI: −60.6, 83.5) at 270 days post-vaccination (Figure 1C), representing 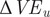 of 56.9% within 9 months of vaccination. Fewer children were available for analyses as the number of days since vaccination increases, diminishing statistical power for VE estimation (Supplementary Figure S1). Bias-corrected VE waned from 93.5% (95% CI: 84.0, 97.3) to 45.8% (95% CI: −46.0, 84.9) (Figure 1C) between 14 and 270 days post-vaccination, corresponding to 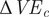 of 51.0% within 9 months, with a 5.9 percentage point overestimation compared to 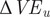 . When waning was assessed up to day 120, uncorrected VE waned from 93.5% (95% CI: 84.0, 97.3) to 83.8% (95% CI: 74.5, 90.2), resulting in 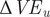 of 10.4% within 4 months of vaccination. Bias-corrected VE was 93.5% (95% CI: 84.0, 97.3) at 14 days and 85.0% (95% CI: 76.4, 90.9) at 120 days post-vaccination, corresponding to 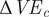 of 9.1% over 4 months, with a 1.3 percentage point overestimation in 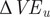 .

In sensitivity analyses assuming VE against infection of 30% to derive infection risk in vaccinated children, bias-corrected VE waned from 93.5% (95% CI: 84.0, 97.3) to 43.3% (95% CI: −52.3, 84.3), corresponding to 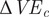 of 53.7% within 9 months post-vaccination and an overestimation of 3.2 percentage points in 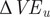 (Supplementary Figure S2A). Assuming VE against infection of 70%, 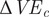 was 49.7% within 9 months post-vaccination with an overestimation of 7.2 percentage points in 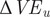 (Supplementary Figure S2B).

Assuming 80% of unvaccinated children were uninfected at the beginning of the season and all vaccinated children (100%) were uninfected significantly overestimated VE (Supplementary Figure S2C). From 14 to 270 days post-vaccination, bias-corrected VE was expected to wane from 94.8% (95% CI: 87.2, 97.9) to 56.6% (95% CI: −16.8, 88.0), with a 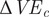 of 40.3% within 9 months, 16.7 percentage points lower than 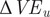 . Conversely, if 80% of vaccinated children and 100% of unvaccinated children were uninfected in the current season at 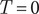 , waning in the uncorrected VE would be underestimated, resulting in 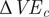 of 64.9% at 9 months post-vaccination (Supplementary Figure S2D), 8.0 percentage points higher than 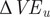 . Varying the proportions of uninfected children at 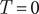 from 0.5 to 1.0 showed minimal bias in uncorrected VE if these proportions were non-differential by vaccination status (Supplementary Figure S3).

The waning rate of uncorrected VE over 9 months would still be overestimated by 2.4 percentage points, even with accurate infection risk estimates (Supplementary Figure S2E). Such overestimation was inflated and could be significant if infection risk was underestimated, leading to 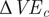 of 45.7%, which was 11.2 percentage points lower than 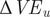 (Supplementary Figure S2F).

## Discussion

Quantifying true waning in VE by time since vaccination poses challenges due to artefacts such as differential depletion of susceptible individuals. We found that the potential impact of such bias on our observed waning rate of VE against influenza A(H1N1)pdm09 was likely minimal. However, if the proportions of individuals remaining uninfected at the time vaccination was offered differed significantly based on vaccination status, the waning rate of uncorrected VE could be over- or underestimated. Our results remained robust unless infection risks were underestimated by a factor of 5, as illustrated in the scenarios explored.

There may be several explanations for our observation that there was minimal bias due to differential depletion of susceptibles, including a high observed VE and low infection risk during the study season. Previous simulation studies have demonstrated that VE analyses are less susceptible to bias due to differential depletion of susceptibles when the baseline VE is high, whether or not VE truly wanes [22, 23]. In our study, influenza vaccination was highly effective against hospitalization due to influenza A(H1N1)pdm09 with an initial estimate of 93.5%. A higher infection risk would drive differential acquisition of infection-induced immunity particularly if VE is also high, thereby introducing a greater degree of bias.

Our current work has highlighted several important considerations when applying the approach of Andrejko et al. [7], which was developed for SARS-CoV-2, to correct for differential depletion-of-susceptibles bias for influenza VE. The approach differentiates the sources of immunity including whether it was acquired through vaccination and natural infection over the course of an epidemic. Such differentiation is, however, not so straightforward for influenza as it has been for SARS-CoV-2. Influenza viruses have been circulating in the community for a much longer period than SARS-CoV-2, and uptake of annual influenza vaccines has generally increased from year to year [24, 25]. Therefore, in influenza VE studies, patients may have more complex history of vaccination and infection, and our assumptions about infection risk could be overestimated. Our study may have avoided this problem to some extent by limiting the analysis to children who would have been exposed to a more limited repertoire of influenza viruses than adults.

The compounding effects of repeated vaccination or natural infection should also be considered [26, 27]. If immunity obtained from the previous season also wanes over time, we would expect an attenuation in the bias due to differential depletion of susceptibles. However, infection-induced immunity probably lasts longer than vaccine-induced immunity in which case the bias may be exacerbated [28]. Repeated vaccination is also well known to complicate the expected immune response and may result in better or worse protection in the current season depending on the influenza types/subtype in question [29]. Without detailed individual-level information on prior influenza exposures, we made a simplified assumption that everyone had an equal chance of infection in the current season regardless of sources of prior exposure. Our sensitivity analyses examining the proportion of individuals remaining uninfected at the time vaccination was offered mimicked a scenario where susceptibility was differential by vaccination status in the current season due to lingering immunity from a prior exposure. In the scenarios explored, VE over time could be biased in either direction, consistent with Andrejko et al.’s findings for SARS-CoV-2 [7].

For both viruses, but especially for influenza viruses, the power of serological assays to discriminate infection from vaccination is limited. As peoples’ immune histories against SARS-CoV-2 increase in complexity due to prior vaccination and infection, it will similarly become difficult to disentangle the confounding effects of these prior exposures on the depletion of susceptibles effect. Controlling for this confounding in TND studies could be remedied through the collection of sera in the acute phase of illness, prior to antibody production, to estimate exposure histories [28, 30] and control for those effects in the analysis, but more research is needed in this area to determine the utility of this approach.

It is important to note that our work specifically examined hospitalizations due to influenza A(H1N1)pdm09 and may not be generalizable to other influenza types or subtypes. Unfortunately, our sample sizes were insufficient to meaningfully assess the extent of bias against influenza A(H3N2) or B. Similarly, our estimates applied to children and may not be applicable to adults or elderly populations, the immune histories for whom will be far more complex. Finally, we only looked at one season. Given the rapid rate of change that influenza viruses undergo, and the frequent updates to the vaccine antigens, data from more seasons will be needed to confirm our findings. It may be the case that in some seasons, potentially those during which waning was particularly prominent, the influence of depletion-of-susceptibles bias may be more prominent.

In conclusion, our work provides some reassurance that our earlier estimates of the rate of waning VE in this paediatric population are not likely to be meaningfully harmed by depletion-of-susceptibles bias and remain valid. Further studies are needed to confirm whether depletion-of-susceptibles bias may have a greater impact across settings, seasons, and age groups. Waning VE remains a problem, and optimizing the timing of vaccination will allow maximal public health impact of influenza vaccination campaigns.

## Supporting information

10.1017/S0950268825100770.sm001 Chua et al. supplementary material Chua et al. supplementary material

## Data Availability

The serological and surveillance data that support the findings of this study are openly available at https://github.com/timktsang/influenza_titer_reconstruction [16] and https://www.chp.gov.hk/en/resources/29/304.html [14]. The TND data are not publicly available due to confidentiality considerations.

## Supplementary material

The supplementary material for this article can be found at http://doi.org/10.1017/S0950268825100770.

## References

[1] Feng S, et al. (2018) Effectiveness of influenza vaccination on influenza-associated hospitalisations over time among children in Hong Kong: A test-negative case-control study. The Lancet Respiratory Medicine 6, 925–934. 10.1016/S2213-2600(18)30419-3. 30442587 PMC6637165

[2] Kissling E, et al. (2018) 2015/16 I-MOVE/I-MOVE+ multicentre case-control study in Europe: Moderate vaccine effectiveness estimates against influenza a(H1N1)pdm09 and low estimates against lineage-mismatched influenza B among children. Influenza and Other Respiratory Viruses 12, 423–437. 10.1111/irv.12520. 29125681 PMC6005601

[3] Belongia EA, et al. (2015) Waning vaccine protection against influenza a (H3N2) illness in children and older adults during a single season. Vaccine 33, 246–251. 10.1016/j.vaccine.2014.06.052. 24962752 PMC7463277

[4] Lipsitch M, et al. (2019) Depletion-of-susceptibles bias in influenza vaccine waning studies: How to ensure robust results. Epidemiology and Infection 147, e306. 10.1017/S0950268819001961. 31774051 PMC7003633

[5] Lewnard JA, et al. (2018) Measurement of vaccine direct effects under the test-negative design. American Journal of Epidemiology 187, 2686–2697. 10.1093/aje/kwy163. 30099505 PMC6269249

[6] Ray GT, et al. (2019) Intraseason waning of influenza vaccine effectiveness. Clinical Infectious Diseases 68, 1623–1630. 10.1093/cid/ciy770. 30204855 PMC7182205

[7] Andrejko KL, et al. (2023) Waning of 2-dose BNT162b2 and mRNA-1273 vaccine effectiveness against symptomatic SARS-CoV-2 infection accounting for depletion-of-susceptibles bias. American Journal of Epidemiology 192, 895–907. 10.1093/aje/kwad017. 36702469 PMC10236522

[8] Centre for Health Protection. (2024) Seasonal influenza vaccination. Available at https://www.chp.gov.hk/en/features/107880.html (accessed 27 March 2025).

[9] Chiu SS, et al. (2016) Hospital-based vaccine effectiveness against influenza B lineages, Hong Kong, 2009-14. Vaccine 34, 2164–2169. 10.1016/j.vaccine.2016.03.032. 27013437

[10] Tsang TK, et al. (2022) Reconstructing antibody dynamics to estimate the risk of influenza virus infection. Nature Communications 13, 1557. 10.1038/s41467-022-29310-8. PMC8943152 35322048

[11] Cowling BJ, et al. (2014) Incidence of influenza virus infections in children in Hong Kong in a 3-year randomized placebo-controlled vaccine study, 2009-2012. Clinical Infectious Diseases 59, 517–524. 10.1093/cid/ciu356. 24825868

[12] Cowling BJ, et al. (2012) Protective efficacy against pandemic influenza of seasonal influenza vaccination in children in Hong Kong: A randomized controlled trial. Clinical Infectious Diseases 55, 695–702. 10.1093/cid/cis518. 22670050

[13] Cowling BJ, et al. (2010) Comparative epidemiology of pandemic and seasonal influenza a in households. The New England Journal of Medicine 362, 2175–2184. 10.1056/NEJMoa0911530. 20558368 PMC4070281

[14] Centre for Health Protection. (2023) Flu Express (Last update for week 4, 2023). Available at https://www.chp.gov.hk/en/resources/29/304.html (Accessed 27 March 2025).

[15] Wu P, et al. (2012) Excess mortality associated with influenza a and B virus in Hong Kong, 1998-2009. The Journal of Infectious Diseases 206, 1862–1871. 10.1093/infdis/jis628. 23045622 PMC3502382

[16] Tsang TK (2023) Influenza titer reconstruction. Available at https://github.com/timktsang/influenza_titer_reconstruction (accessed 15 October 2024).

[17] Cauchemez S, et al. (2012) Influenza infection rates, measurement errors and the interpretation of paired serology. PLoS Pathogens 8, e1003061. 10.1371/journal.ppat.1003061. 23271967 PMC3521724

[18] Hood N, et al. (2023) Influenza vaccine effectiveness among children: 2011-2020. Pediatrics 151. 10.1542/peds.2022-059922. PMC10071433 36960655

[19] Campbell AP, et al. (2020) Vaccine effectiveness against pediatric influenza hospitalizations and emergency visits. Pediatrics 146. 10.1542/peds.2020-1368. 33020249

[20] Bond HS, Sullivan SG and Cowling BJ (2016) Regression approaches in the test-negative study design for assessment of influenza vaccine effectiveness. Epidemiology and Infection 144, 1601–1611. 10.1017/S095026881500309X. 26732691 PMC5545127

[21] Vehtari A, Gelman A and Gabry J (2017) Practical Bayesian model evaluation using leave-one-out cross-validation and WAIC. Statistics and Computing 27, 1413–1432. 10.1007/s11222-016-9696-4.

[22] Kahn R, et al. (2022) Identifying and alleviating bias due to differential depletion of susceptible people in postmarketing evaluations of COVID-19 vaccines. American Journal of Epidemiology 191, 800–811. 10.1093/aje/kwac015. 35081612 PMC8807238

[23] Kahn R, et al. (2024) Examining bias from differential depletion of susceptibles in vaccine effectiveness estimates in settings of waning. American Journal of Epidemiology 193, 232–234. 10.1093/aje/kwad191. 37771045 PMC10773472

[24] McGovern I, et al. (2022) Influenza vaccine uptake in the United States before and during the COVID-19 pandemic. Vaccines (Basel) 10. 10.3390/vaccines10101610. PMC9612058 36298475

[25] Chua H, et al. (2021) Influenza vaccine effectiveness against influenza-associated hospitalization in children in Hong Kong, 2010-2020. Vaccine 39, 4842–4848. 10.1016/j.vaccine.2021.07.014. 34301433

[26] Krammer F (2019) The human antibody response to influenza a virus infection and vaccination. Nature Reviews Immunology 19, 383–397. 10.1038/s41577-019-0143-6. 30837674

[27] Hoa LNM, et al. (2022) Influenza a(H1N1)pdm09 but not a(H3N2) virus infection induces durable seroprotection: Results from the Ha Nam cohort. The Journal of Infectious Diseases 226, 59–69. 10.1093/infdis/jiaa293. 32484513 PMC9373157

[28] Fox A, et al. (2022) Opposing effects of prior infection versus prior vaccination on vaccine immunogenicity against influenza a(H3N2) viruses. Viruses 14. 10.3390/v14030470. PMC8949461 35336877

[29] Jones-Gray E, et al. (2023) Does repeated influenza vaccination attenuate effectiveness? A systematic review and meta-analysis. The Lancet Respiratory Medicine 11, 27–44. 10.1016/S2213-2600(22)00266-1. 36152673 PMC9780123

[30] Hay JA, et al. (2024) Reconstructed influenza a/H3N2 infection histories reveal variation in incidence and antibody dynamics over the life course. PLoS Biology 22, e3002864. 10.1371/journal.pbio.3002864. 39509444 PMC11542844

