# Supplementary figures and images for: Waning in influenza vaccine effectiveness against influenza A(H1N1)pdm09-associated hospitalization in children in 2012/2013
Source: Epidemiol Infect. 2025 Nov 24;153:e137. doi: 10.1017/S0950268825100770 (PMC12722548; doi:10.1017/S0950268825100770)

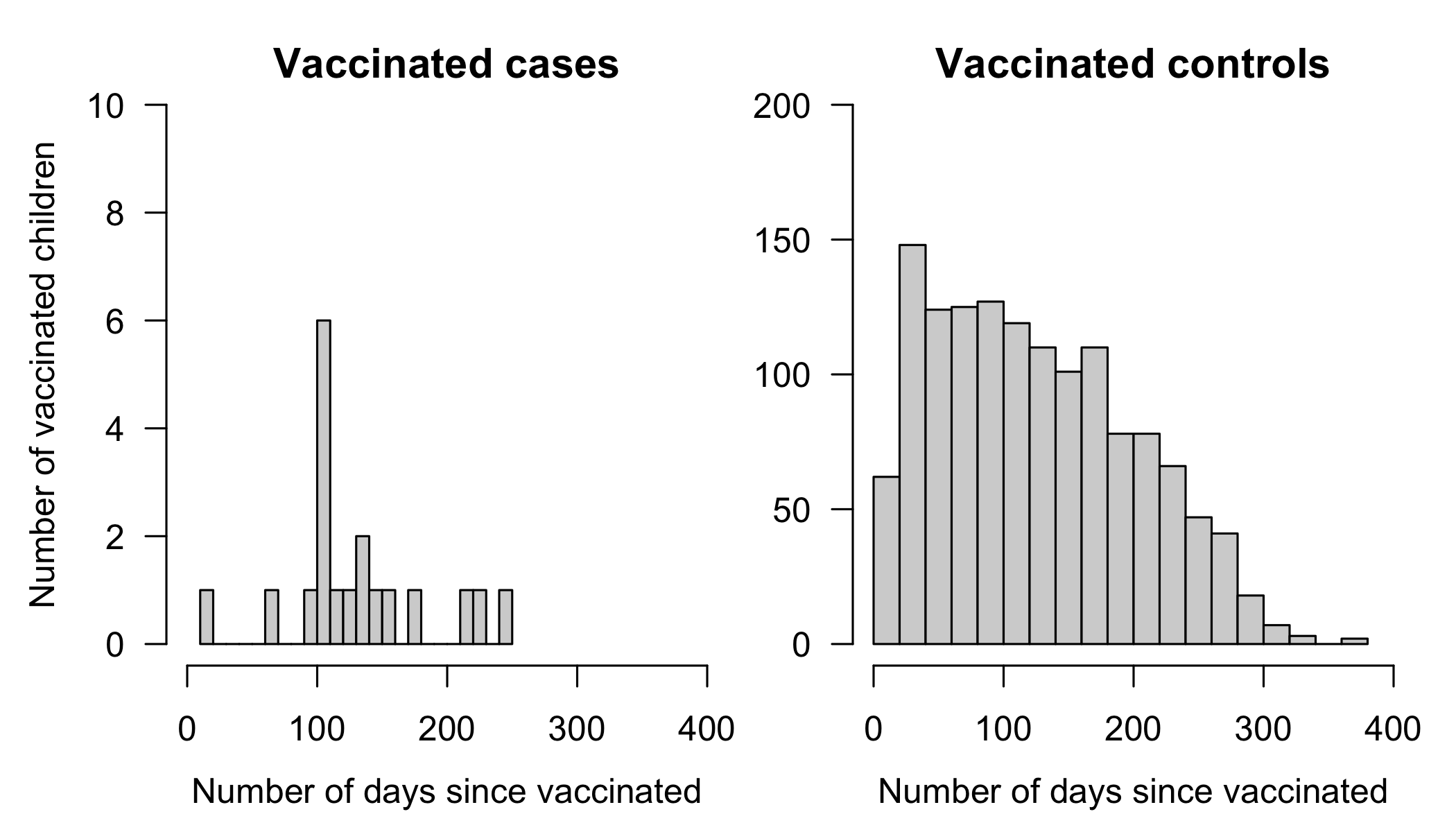

Supplement: Chua et al. supplementary material [file S0950268825100770sup001.zip › S0950268825100770sup001.tiff]
